# Synthesis and Inhibiting Activity of Some 4-Hydroxycoumarin Derivatives on HIV-1 Protease

**DOI:** 10.5402/2011/137637

**Published:** 2011-07-26

**Authors:** Stancho Stanchev, Frank Jensen, Anton Hinkov, Vasil Atanasov, Petia Genova-Kalou, Radka Argirova, Ilia Manolov

**Affiliations:** ^1^Department of Chemistry, Faculty of Pharmacy, 2 Dunav Street, 1000 Sofia, Bulgaria; ^2^Department of Chemistry, Faculty of Science, University of Aarhus, Langelandsgade 140, 8000 Aarhus C, Denmark; ^3^Laboratory of Virology, Faculty of Biology, Sofia University “St. Kliment Ohridski”, 8 Dragan Zankov, 1164 Sofia, Bulgaria; ^4^Laboratory of Biocoordination and Bioanalytical Chemistry, Faculty of Chemistry, Sofia University “St. Kliment Ohridski”, 1 J. Bourchier, 1164 Sofia, Bulgaria; ^5^Laboratory of Cell Cultures, National Center of Infectious and Parasitic Diseases, 44 A Stoletov Street, 1233 Sofia, Bulgaria; ^6^Laboratory of Retroviruses, National Center of Infectious and Parasitic Diseases, 44 A Stoletov Street, 1233 Sofia, Bulgaria; ^7^Department of Pharmaceutical Chemistry, Faculty of Pharmacy, 2 Dunav Street, 1000 Sofia, Bulgaria

## Abstract

Six novel 4-hydroxycoumarin derivatives were rationally synthesized, verified, and characterized by molecular docking using crystal HIV-1 protease. Molecular docking studies predicted antiprotease activity of **(7)** and **(10)**. The most significant functional groups, responsible for the interaction with HIV-1 protease by hydrogen bonds formation are pyran oxygen, atom, lactone carbonyl oxygen and one of the hydroxyl groups. The newly synthesized compounds were biologically tested in MT-4 cells for inhibiting HIV-1 replication, exploring the protection of cells from the cytopathic effect of HIV measured by cell survival in MTT test. One derivative −**7** showed 76–78% inhibition of virus infectivity with IC_50_ = 0.01 nM, much less than the maximal nontoxic concentration (1 mM). Antiprotease activity of **7** in two different concentrations was detected to be 25%. Nevertheless, the results of study of **(7)** encourage using it as a pharmacophore for further synthesis and evaluation of anti-HIV activity.

## 1. Introduction

The retroviral protease (PR) of human immunodeficiency virus type 1 (HIV-1) is one of the key enzymes for virus replication. It cleaves protein and glycoprotein precursors to yield active viral enzymes and structural proteins. The inactive HIV-1 PR leads to noninfectious virions. This fact stimulated search of potent substances with antiprotease activity inhibiting HIV-1 replication. During the past 12 years, a number of peptidomimetic analogs—inhibitors of HIV-1 PR (PIs)— have been clinically introduced, but the largest part of them show poor pharmacological characteristics such as bad oral bioavailability, rapid clearance, and tolerability problems—often associated with lypodystrophy and dyslipidemia [1]. Also, because of being peptidomimetics, viral isolates quickly demonstrate a high degree of resistance and cross-resistance even when using the members of the group before PIs were put on the market [2].

**Figure 1:**
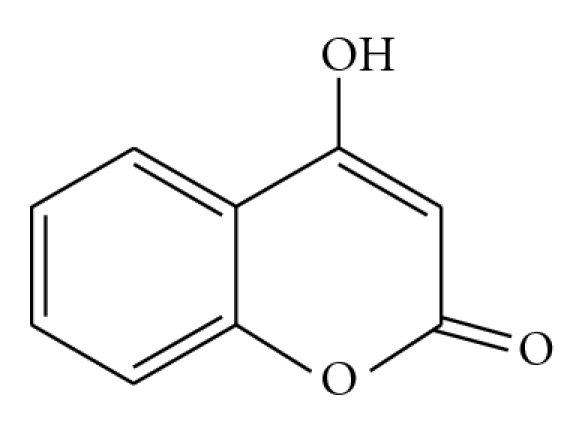
Structure of 4-hydroxycoumarin.

Development of new nonpeptidic PIs, such as Tipranavir and Darunavir, showed an impressive potency against PI-resistant mutants, so remaining an important option for patients harboring such resistance [3]. This is the reason to search for novel nonpeptidic substances—inhibitors of HIV-1 protease. Experimental data on some nonpeptidic substances—4-hydroxycoumarins (Figure 1) and 4-hydroxypyran derivatives inhibiting HIV-1 PR, support this idea [4]. 

Being for long years interested in synthesis and evaluation of a range of nonpeptidic substances such as 4-hydroxycoumarin derivatives [5–9], we were encouraged to extend these experiments. Here, a number of new syntheses are presented, accompanied with molecular docking experiments using crystal enzyme and biological activity evaluation of novel and promising 4-hydroxycoumarin derivatives. 

## 2. Results and Discussion

### 2.1. Synthesis of 4-Hydroxycoumarin Derivatives

#### 2.1.1. Synthesis of Arylmethylene-*β*-Ketoesters [10]

**Figure 2:**
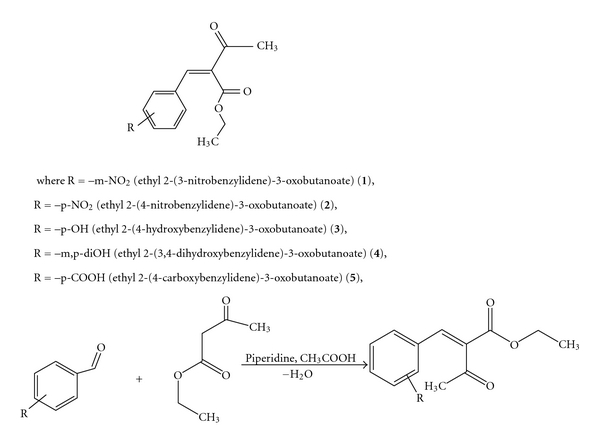
General chemical structure and synthesis of arylmethylene-*β*-ketoesters.

Differently substituted aromatic aldehydes are used for synthesis of arylmethylene-*β*-ketoesters via Knoevenagel reaction with ethyl acetoacetate in the presence of piperidine as a basic agent and glacial acetic acid. These arylmethylene-*β*-ketoesters may be presented as in Figure 2. 

**Figure 3:**
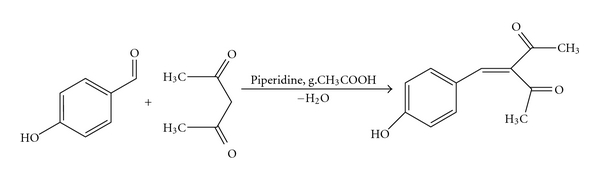
Synthesis of 3-(4-hydroxy)phenylmethylene-2,4-pentanedione (**6**).

A reaction of 4-hydroxybenzaldehyde and 2,4-pentanedione was also performed in the same conditions as above mentioned. The isolated product was 3-(4-hydroxy)phenylmethylene-2,4-pentanedione (**6**) [11], see Figure 3.

#### 2.1.2. The Michael Addition of Arylmethylene-*β*-Ketoesters and Arylmethylene-2,4-Pentanediones to 4-Hydroxycoumarin

**Figure 4:**
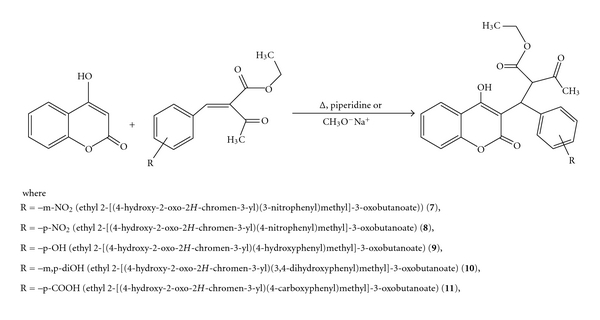
The Michael addition of 4-hydroxycoumarin and arylmethylene-*β*-ketoesters.

The second step of the reaction is an addition of the obtained arylmethylene-*β*-ketoesters with 4-hydroxycoumarin through the Michael reaction, by using sodium methoxide or piperidine as a basic agent [10]. This reaction can be expressed as in Figure 4.

**Figure 5:**
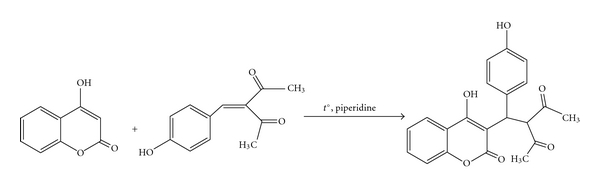
The Michael addition of 4-hydroxycoumarin and 3-(4-hydroxy)phenylmethylene-2,4-pentanedione (**6**).

3-(4-Hydroxy)phenylmethylene-2,4-pentanedione reacted with 4-hydroxycoumarin at the refluxing and the presence of piperidine. The product of the Michael addition was 3-[(4-hydroxy-2-oxo-2*H*-chromen-3-yl)(4-hydroxyphenyl)methyl]pentane-2,4-dione (**12**) [11], see Figure 5. 

### 2.2. Molecular Docking

The interaction of HIV-1 protease by molecular docking with some new synthesized 4-hydroxycoumarin derivatives was investigated. Experimental data for the activity of some 4-hydroxycoumarins were used for comparison. 

Preliminary molecular docking was made based on known 4-hydroxycoumarin derivatives, which have inhibiting activity of HIV-1 protease (Table 1) [4]. The grid is chosen to oversize the ligand which is previously bound to the enzyme (in this case 7 Å size of the grid was chosen). 

The *G*-score and E-model functions values have been obtained by this method. They are called scoring function and they are abstract equivalents of Δ*G*_bind_. These functions take into account the free energy due to the solvent effects, conformational changes in protein and the ligand, interactions between protein and the ligand (hydrogen bonds, ionic interaction, and van der Waals forces), free energy loss of freezing of internal rotation of protein and the ligand, free energy loss in translational and rotational energy, caused by association of the two molecules, and the free energy due to changes in vibrational mode (usually ignored). If these values are more negative for one ligand, this means that the binding capability of this ligand is better. When the ligand shows binding capability in one determined conformation, the program shows this as a “good pose”. 

This enzyme consists of two polypeptide chains and it is from the aspartyl protease family with two aspartate residues lying at the bottom of the active site. We used crystallographic structure of the retroviral HIV-1 protease, bound with a peptidomimetic inhibitor BEA369 from Protein Data Bank with pdb code 1EBY. 

It can be concluded that ligand (**1**) has the strongest binding capability to HIV-1 protease. The results of molecular docking confirm the experimental data about ligand (**1**).

Concerning ligands (**1**)–(**5**), the *G*-score and E-model values from molecular docking, for the group of tested compounds, are shown in Table 2.

The interactions between the investigated 4-hydroxycoumarin derivatives and the active sites of the enzyme HIV-1 protease are realized by hydrogen bonding and the van der Waals interactions. The most active compound for example, with the best binding activity, is compound (**10**), according ligands (**1**)–(**4**) (based on the values of the scoring functions). This fact probably dues to a formation of hydrogen bonds between pyran oxygen atom, lactone carbonyl oxygen, and one of the hydroxyl groups (in metaposition) attached to the aromatic ring from the side chain. The van der Waals forces of attraction also contribute for a good binding. According to ligand (**5**), the most active is compound (**7**). There are two hydrogen bonds for compound (**7**) with participation of pyran oxygen atom, carbonyl oxygen atom from the lactone ring and one and corresponding protein fragments. The van der Waals interactions of attraction, in which probably m-nitro group is participating with one of its oxygen atoms are substantial to the binding. The compound (**7**) seems to be more active than the experimental ligands.

### 2.3. Biological Activity in Cell Culture (MT-4 Cells)

After demonstrating the activity of some 4-hydroxycoumarin derivatives towards isolated HIV-PR in molecular docking experiments, it would be of interest to further test them on HIV-1-infected MT-4 cells. The evaluation of anti-HIV effect was done by an *in vitro *rapid and sensitive microtiter infection assay based on cytolysis quantitation by vital dye (MTT) uptake as an endpoint for infection [12]. Additionally, the effect of inhibitors on endogenous reverse transcriptase (RT) activity of HIV-1 III B-infected MT-4 supernatants was considered as a marker for the ability of blocking HIV-1 replication. MT-4 cells were infected and incubated with each inhibitor for 72–96 hours, and then RT activity was measured in the cell supernatants according to guidelines in the HS-Lenti Kit-RT assay (Cavidi, Sweden). A study of direct effect of the newly synthesized 4-hydroxycoumarins on exogenous recombinant RT (rRT) was also performed. Further, all the compounds were tested for anti-HIV-1 PR activity. Table 3 represents the results of microtiter infection assay using MTT and inhibition of HIV-1 PR activity. The experiments were carried out in maximal nontoxic concentration (MNC) for each compound.

First of all, it is seen that the compounds (**8**), (**7**), and (**12**) have higher MNC meaning they are more cytotoxic than the other three compounds. Only two of them (**10**) and (**7**) inhibited viral replication in MT-4 cells, the inhibition induced by (**7**) was remarkable (78%). Using 10x viral dilutions, IC_50_ was established to be 0.01 nM. No compound showed effect on both endogenous and exogenous RT. This means that RT was not the target of the antiviral action. As predicted by molecular docking studies, HIV-1 PR activity was inhibited 24-25% were by (**7**) (5 separate evaluations were done). The discordance found for (**7**) between the data concerning inhibition of infectivity (about 75%) and protease activity (25%) could be explained by another activity, such as l anti-integrase one. It is well known that some 4-hydroxycoumarin derivatives are integrase inhibitors. 

The experiments described in this paper expand the earlier reported ones that 4-hydroxycoumarin derivatives could serve as novel nonpeptidic PIs. Similarly to tipranavir and darunavir, they could be effective in patients with developed resistance to peptidic PIs. Especially, (**7**) could further be used as a pharmacophore to synthesize new more active derivatives towards HIV-1 protease. 

## 3. Conclusion

Six 4-hydroxycoumarin compounds were synthesized by two-step synthesis. First step is the Knoevenagel reaction between aromatic aldehydes and ethyl acetoacetate or acetylacetone. The second step is the Michael addition of the obtained arylmethylene-*β*-ketoester or arylmethylene-2,4-pentanedione with 4-hydroxycoumarin. The products are identified and characterized by ^1^H NMR, EI-MS, FTIR, and element analysis.

Studying their binding activity to HIV-1 PR was performed by molecular docking. The crystal HIV-1 PR, bound with peptidomimetic inhibitor BEA369, was used. The highest binding activity is showing compound (**10**), according to the experimental ligands (**1**), (**2**), (**3**), and (**4**) and compound (**7**), according to ligand 5. This fact probably is due to a formation of hydrogen bonds between pyran oxygen atom, lactone carbonyl oxygen, and one of the hydroxyl groups (in metaposition) attached to the aromatic ring from the side chain. The van der Waals forces of attraction also contribute to a good binding.

All six compounds were tested for anti-HIV-1 PR activity in MT4 cells infected by HIV-1. The cell survival was evaluated by MTT test and also the % of HIV-1 PR inhibition was measured. The highest inhibition of HIV-1 PR (25%) and highest MT4 cell survival (78%) were demonstrated by compound (**7**). Compound (**7**) could further be used as a pharmacophore to synthesize new more active derivatives towards HIV-1 PR.

## 4. Experimental Part

### 4.1. Synthesis of 4-Hydroxycoumarin Derivatives

#### 4.1.1. Materials and Methods

All starting materials were purchased from Merck, Sigma-Aldrich, and Fluka. They are used without further purification. Melting points are measured in open capillary tubes on a Büchi 535 melting point apparatus. The IR spectra were recorded at Shimadzu FT-IR 8101 M spectrometer in nujol, and frequencies are expressed in cm^−1^. The ^1^H NMR spectra were recorded in Brucker 250 MHz in DMSO-d_6_ or acetone using TMS as an internal standard (chemical shifts are reported in ppm units, coupling constants (*J*) in Hz). Abbreviations are as follows: s: singlet, d: doublet, dd: double doublet, dq: double quartet, dqui: double quintet, t: triplet, and m: multiplet.

Mass-spectral analysis was performed by electron ionization on masspectrometer Hewlett-Packard 5973 at 70 eV.

#### 4.1.2. General Procedure for the Preparation of Arylmethylene-*β*-Ketoesters

Aromatic aldehyde and ethylacetoacetate in equimolar quantities are mixed in a round-bottomed flask. Piperidine (0.03 mol) and glacial acetic acid (0.04 mol) are also added to the reaction mixture. The latter is stirred at room temperature for 90 minutes. After 20 mL ether and/or 150 mL distilled water are added to the reaction mixture, the crystals with different colors are formed. These crystals are filtered and washed. Then, they are dried at room temperature and recrystallized in appropriate solvents—mainly alcohols (ethanol, propanol, and 2-propanol) and water.

**Physical and Spectral Data**

**Ethyl 2-(3-Nitrobenzylidene)-3-Oxobutanoate (1) [10]**

White crystals, m.p. 100–103°C. The substance crystallizes from water. Purified after recrystallization from isopropyl alcohol. Yield: 17%. UV-VIS: *λ*_max⁡_ = 210, 266 nm; FTIR (nujol): 1728.4, 1660.9, 1628.1, 1529.7, 780, 735 cm^−1^; ^1^H NMR (DMSO, 250 MHz): *δ* = 0.9 (t, *J* = 7.1 Hz, 3H) (methyl), 1.6 (s, 3H) (acetyl), 4.8 (q, *J* = 7.1 Hz, 2H) (methylene), 7.09–7.02 (m, 1H) (aromatic), 7.58–7.56 (m, 1H) (methyne), 7.92–7.87 (m, 1H) (aromatic), 8.36–8.31 (m, 1H) (aromatic), 8.66–8.64 (m, 1H) (aromatic); EIMS: m/z (%) = 263 (65.2, M^+^), 262 (20), 248 (99.1), 246 (100), 234 (19.1), 220 (32.1), 218 (51.8), 216 (15.7), 202 (35.7), 200 (24.3), 192 (13), 180 (18.3), 176 (66.09), 174 (27), 160 (10.4), 152 (21.7), 146 (13), 130 (20.9), 129 (36.5), 120 (17.4), 115 (19.1), 102 (35.7), 101 (47.8), 89 (13.9), 75 (29.6), 63 (9.6), 51 (13), 45 (2.6); TLC: *R*_*f*_ = 0.5 (hexane/acetone = 2 : 1); Anal.: C_13_H_13_NO_5_, (263), (C, H) (calcd/found): % C 59.31/59.54, % H 4.98/H 5.13.

**Ethyl 2-(4-Nitrobenzylidene)-3-Oxobutanoate (2) [10]**

White crystals. m.p. 160-161°C. The substance crystallizes from ether. Purified after recrystalization from isopropyl alcohol; Yield: 66%; UV-VIS: *λ*_max⁡_ = 204, 270 nm; FTIR (nujol): 1732.3, 1711.1, 1608.8, 1529.7, 1464.1, 844.9 cm^−1^; ^1^H NMR (Acetone, 250 MHz): *δ* = 0.9 (t, *J* = 7.1 Hz, 3H) (methyl), 1.3  (s, 3H) (acetyl), 2.8 (q, *J* = 7.1 Hz, 2H) (methylene), 6.1 (s, 1H) (methyne), 7.68–7.62 (m, 2H) (aromatic), 7.95–7.89 (m, 2H) (aromatic); ^13^C NMR (Acetone, 67 MHz): *δ* = 15, 30, 45, 125, 130, 145, 165, 175, 200; TLC: *R*_*f*_ = 0,75 (hexane/chloroform/acetone/methanol = 5 : 3 : 2 : 1), Anal.: C_13 _H_13 _NO_5_ (263), (C, H) = (calcd/found): % C 59.31/58.28, % H 4.98/5.92, % N 5.32/3.52.

**Ethyl 2-(4-Hydroxybenzylidene)-3-Oxobutanoate (3) [13–16]**

Yellow crystals, m.p 141–143°C. The substance crystallizes from ether. Purified after recrystallization from isopropyl alcohol. Yield: 51%; UV-VIS: *λ*_max⁡_ = 206, 224, 286 nm, FTIR (nujol): 3325.7, 1732.3, 1641.6, 1597.3, 1462.2, 1205.7, 819.8 cm^−1^; ^1^H NMR (Acetone, 200 MHz): *δ* = 1.3 (t, *J* = 7.1 Hz, 3H) (methyl), 2.3 (s, 3H) (acetyl), 4.3 (q, *J* = 7.1 Hz, 2H) (methylene), 6.91–6.85 (m, 2H) (aromatic), 7.44–7.38 (m, 2H) (aromatic), 7.46–7.44 (m, 1H) (methyne), 10.5 (s, 1H) (hydroxyl).^ 13^C NMR (Acetone, 67 MHz): *δ* = 30, 110, 135, 140, 160, 190; EIMS: m/z (%) = 234 (100, M^+^), 233 (57), 220 (10), 219 (69), 217 (17), 205 (15), 191 (25.4), 189 (38.25), 187 (11.3), 175 (8.7), 163 (11.3), 161 (11.3), 160 (28.7), 151 (28.7), 147 (68.7), 146 (11.3), 145 (37.4), 131 (7), 123 (30.4), 120 (9.6), 119 (20), 118 (19.1), 115 (2.6), 107 (6), 91 (20), 89 (19.1), 77 (7), 65 (11.3), 63 (12.1), 53 (5), 45 (0.9); TLC: *R*_*f*_ = 0.39 (hexane/acetone = 2 : 1); Anal.: C_13 _H_14 _O_4_, (234), (C, H) = (calcd/found): % C 66.66/66.50, % H 6.02/5.94.

**Ethyl 2-(3,4-Dihydroxybenzylidene)-3-Oxobutanoate (4) [17]**

Brown-yellow crystals, m.p. 147.8–151°C. The substance crystallizes from water. Purified after recrystallization from water. Yield: 19%; UV-VIS: *λ*_max⁡_ = 206, 252, 344 nm; FTIR (nujol): 3540, 1714.9, 1643.6, 1603, 1464.1, 1197 cm^−1^; ^1^H NMR (DMSO, 250 MHz): *δ* = 1.0 (t, *J* = 7.1 Hz, 3H) (methyl), 2.3 (s, 3H) (acetyl), 4.2 (q, *J* = 7.1 Hz, 2H) (methylene), 6.91 (dq, *J* = 8.13 Hz, 1H) (aromatic), 7.0 (dq, *J* = 8.13 Hz, 1H) (aromatic), 7.14–7.13 (m, 1H) (aromatic), 7.44–7.42 (m, 1H) (methyne), 8.4 (s, 2H) (hydroxyl); EIMS: m/z (%) = 250 (100, M^+^), 249 (40), 235 (20), 233 (29.6), 222 (17.4), 205 (31), 189 (76.5), 177 (10.4), 176 (31.3), 163 (42.6), 161 (73.9), 147 (4.3), 134 (19.1), 117 (9.6), 103 (5.2), 89 (15.7), 88 (11.3), 77 (11.3), 69 (4.3), 62 (8.7), 51 (8.7); TLC: *R*_*f*_ = 0.4 (hexane/chloroform/acetone/methanol = 5 : 3 : 2 : 1); Anal.: C_13 _H_14 _O_5_ (250), (C, H) (calcd/found): % C 62.39/62.31, % H 5.64/5.64.

**4-[2-(Ethoxycarbonyl)-3-Oxobut-1-en-1-yl]Benzoic Acid (5) [10]**

Yellow crystals, m.p. 148–150°C. The substance crystallizes from water. Purified after recrystallization from ethanol; Yield: 57%; UV-VIS: *λ*_max⁡_ = 204, 292 nm; FTIR (nujol): band 3300–2400, 1736.1, 1689.8, 1608.8, 1460.3, 848 cm^−1^; ^1^H NMR (DMSO, 250 MHz): *δ* = 1.0 (t, *J* = 7.1 Hz, 3H) (methyl), 2.4 (s, 3H) (acetyl), 4.2 (q, *J* = 7.1 Hz, 2H) (methylene), 7.41–7.39 (m, 1H) (methyne), 7.59–7.53 (m, 2H) (aromatic), 7.96–7.91 (m, 2H) (aromatic), 13.23 (s, 1H) (carboxylic); EIMS: m/z (%) = 262 (64, M^+^), 261 (16.7), 247 (18.4), 233 (8), 218 (18.4), 217 (100), 191 (10), 189 (14.9), 179 (9.6), 175 (26.3), 173 (27.2), 171 (11.4), 155 (14.9), 151 (16.7), 147 (7.9), 131 (9.6), 129 (17.5), 115 (7.9), 103 (18.4), 101 (14.9), 91 (5.3), 77 (11.4), 75 (8.7), 63 (3.5), 51 (2.6), 45 (1.8); TLC: *R*_*f*_ = 0.33 (hexane/chloroform/acetone/methanol = 5 : 3 : 2 : 1); Anal.: C_14 _H_14 _O_5_ (262), (C, H) (calcd/found): % C 64.12/64.44, % H 5.38/5.26.

#### 4.1.3. Procedure for the Preparation of 3-(4-Hydroxy)-Phenylmethylene-2,4-Pentandione (6)

4-Hydroxybenzaldehyde (3.66 g, 0.03 mol) and acetylacetone (5.14 mL, 0.05 mol) are mixed in round-bottomed flask. Piperidine (0.03 mol) and glacial acetic acid (0.04 mol) are also added to the reaction mixture. The latter is stirred at room temperature for 120 minutes. After 150 mL distilled water are added to the reaction mixture. Crystals with different colors are formed. These crystals are filtered off and washed. Then they are dried at room temperature and recrystallized in methylene chloride.

**Physical and Spectral Data**

**3-(4-Hydroxybenzylidene)-2,4-Pentanedione (6) [11]**

Yellow-orange crystals, m.p. 126–129°C. Yield: 22%, FTIR (nujol): 3180, 1705, 1640, 1600, 1580, 1465, 1160, 900, 730 cm^−1^; ^1^H NMR (DMSO, 250 MHz): *δ* = 2.2 (s, 3H) (acetyl), 2.4(s,3H) (acetyl), 4.8 (s, 1H) (methyne), 6.8–6.74 (m, 2H) (aromatic), 7.11–7.06 (m, 2H) (aromatic), 10.2 (s, 1H) (hydroxyl); EIMS: m/z (%) = 204 (100, M^+^), 189(100), 171 (18.5), 161 (100), 147 (100), 133 (9.2), 119 (74), 105 (6.7), 91(38.7), 77 (15.1), 63 (26), 51 (11.8), 45 (0.8); TLC: *R*_*f*_ = 0.55 (hexane/chloroform/acetone/methanol = 5 : 3 : 2 : 1), Anal.: C_12_H_12_O_3_ (204) (C, H) = (calcd/found): % C 70.57/69.92, % H 5.92/5.85.

#### 4.1.4. General Procedure for the Preparation of Condensation Products with 4-Hydroxycoumarin

Arylydene-*β*-ketoester, obtained in previous reaction, and 4-hydroxycoumarin are mixed in equimolar quantities in 25–30 mL methanol (used as a solvent). Sodium methoxide (0.003 mol) as a basic agent is also added to the reagents. The reaction mixture is boiled and stirred for 60 hours under reflux. The reaction is controlled by TLC (hexane : acetone = 2 : 1 or hexane : acetone : chloroform : methanol = 5 : 3 : 2 : 1). When the quantities of reagents are depleted, the heating was stopped. The residue from the reaction mixture was filtered off and was washed with hot water, in order to remove to the 4-hydroxicoumarin which was not reacted. After that, the residue is dried at room temperature and recrystallized in appropriate solvent (methanol, ethanol, or 2-propanol).

**Physical and Spectral Data**

**Ethyl 2-[(4-Hydroxy-2-Oxo-2H-Chromen-3-yl)(3-Nitrophenyl)Methyl]-3-Oxobutanoate (7) [10]**

White crystals, m.p.135–140°C. Purified after recrystallization from ethanol. Yield: 37% UV-VIS: *λ*_max⁡_ = 210 nm; FTIR (nujol): 3335, 1732.3, 1674.4, 1620.4, 1529.7, 1068.7, 763, 736 cm^−1^; ^1^H NMR (DMSO, 250 MHz): *δ* = 1.0 (t, *J* = 6.83 Hz, 3H) (methyl), 1.9 (s, 3H) (acetyl), 3.9 (q, *J* = 6.83 Hz, 2H) (methylene), 4.68–4.61 (m, 2H) (methyne), 7.23–7.17 (m, 1H) (aromatic), 7.4 (dq, *J* = 7.83 Hz, 1H) (aromatic), 7.52–7.46 (m, 1H) (aromatic), 7.64–7.60 (m, 1H) (aromatic), 7.76–7.72 (m, 1H) (aromatic), 7.83–7.77 (m, 1H)(aromatic), 7.86–7.84 (m, 1H)(aromatic), 7.98–7.94 (m, 1H) (aromatic) 9.8 (s, 1H) (hydroxyl); EIMS: m/z (%) = 425 (4.4, M^+^), 382 (4.4), 361 (12.3), 336 (38.6), 320 (1.8), 308 (2.6), 294 (58.8), 278 (100), 266 (8.8), 257 (14.9), 249 (48.2), 248 (91.2), 239 (1.8), 220 (8.8), 205 (1.8), 176 (3.5), 165 (10.5), 139 (5.3), 130 (15.8), 120 (71.9), 101 (13.2), 92 (68.4), 85 (18.4), 75 (14.9), 64 (17.5), 51 (6); TLC: *R*_*f*_ = 0.34 (hexane/acetone = 2 : 1).

**Ethyl 2-[(4-Hydroxy-2-Oxo-2H-Chromen-3-yl)(4-Nitrophenyl)Methyl]-3-Oxobutanoate (8) [10]**

White crystals, m.p. 250–254°C. Purified after recrystallization from ethanol. Yield: 33%; UV-VIS: *λ*_max⁡_ = 206, 272 nm; FTIR (nujol): 3362.3, 1732.3, 1651.3, 1616.5, 1601.1, 833.3, 765.1 cm^−1^; ^1^H NMR (DMSO, 250 MHz): *δ* = 1.0 (t, *J* = 6.83 Hz, 3H) (methyl), 2.0 (s,3H) (acetyl), 3.9 (q, *J* = 6.83 Hz, 2H) (methylene), 4.40–4.36 (m, 1H) (methyne), 4.62 (dq, *J* = 4.25 Hz, 1H) (methylene), 7.25–7.17 (m, 3H) (aromatic), 7.38 (dq, *J* = 7.83 Hz, 1H) (aromatic), 7.51–7.46 (m, 1H) (aromatic), 7.83–7.77 (m, 1H) (aromatic), 7.98–7.94 (m, 1H) (aromatic), 10.0 (s, 1H) (hydroxyl); EIMS: m/z (%) = 426 (0.8, M^+^), 380 (0.8), 368 (0.4), 343 (0.8), 327 (5.3), 317 (3.5), 302 (1.8), 284 (0.9), 274 (0.8), 256 (7), 242 (2.6), 230 (6.1), 213 (27.2), 202 (4.4), 187 (4.4), 176 (5.3), 163 (10), 162 (80.7), 149 (7.9), 134 (1.8), 121 (48.2), 120 (100), 105 (4.4), 92 (57), 77 (7.9), 63 (18.4), 51(6.1), 46 (1.8); TLC: *R*_*f*_ = 0.48 (hexane/acetone = 2 : 1); Anal.: C_22 _H_19 _NO_8_ (426), (C, H) (calcd/found): % C 62.12/62.02, % H 4.5/4.38, % N 3.29/3.21.

**Ethyl 2-[(4-Hydroxy-2-Oxo-2H-Chromen-3-yl)(4-Hydroxyphenyl)Methyl]-3-Oxobutanoate (9) [10]**

White crystals, m.p. 195–197°C. Purified after recrystalisatyion from ethanol. Yield: 21% UV-VIS: *λ*_max⁡_ = 214, 280, 308 nm; FTIR (nujol): 3391.3, 1699.5, 1622.3, 1599.2, 1464.1, 821, 760 cm^−1^; ^1^H NMR (DMSO, 250 MHz): *δ* = 1.0 (t, *J* = 6.83 Hz, 3H) (methyl), 2.0 (s, 3H) (acetyl), 4.1 (q, *J* = 6.83 Hz, 2H), 4.40–4.36 (m, 1H) (methyne), 4.62 (dq, *J* = 4.25 Hz, 1H) (methylene), 6.87–6.82 (m, 2H) (aromatic), 7.23–7.17 (m, 1H) (aromatic), 7.4 (dq, *J* = 7.83 Hz, 1H) (aromatic), 7.55–7.49 (m, 2H) (aromatic), 7.83–7.77 (m, 1H) (aromatic), 7.98–7.94 (m, 1H) (aromatic), 8.6 (s, 2H) (hydroxyl). EIMS: m/z (%) = 396 (0.09, M^+^), 364 (0.09), 350 (0.09), 321 (0.09), 307 (0.9), 279 (0.4), 266 (56.1), 265 (100), 249 (31.6), 237 (10.5), 221 (2.6), 210 (2.6), 181 (1.8), 165 (1.8), 153 (2.6), 146 (7), 130 (7), 121 (19.3), 118 (17.5), 102 (4.4), 92 (15.8), 85 (12.3), 76 (2.6), 63 (10.5), 53 (2.6), 46 (0.09); TLC: *R*_*f*_ = 0.48 (hexane/chlorophorm/acetone/methanol); Anal.: C_22 _H_20 _O_7 _ (396), (C, H) (calcd/found): % C 66.66/66.36, % H 5.09/5.13.

**Ethyl 2-[(3,4-Dihydroxyphenyl)(4-Hydroxy-2-Oxo-2*H*-Chromen-3-yl)Methyl]-3-Oxobuta-Noate (10) [10]**

Tiled-red crystals, m.p. 243.4–247°C. Purified after recrystalization from isopropyl alcohol. Yield: 5%; UV-VIS: *λ*_max⁡_ = 208, 280 nm; FTIR (nujol): 3451, 1732.3, 1662.8, 1608.8, 1460.3, 1180.6, 1109.2, 825.6, 756.2 cm^−1^; ^1^H NMR (DMSO, 250 MHz): *δ* = 1.2 (t, *J* = 6.83 Hz,3H) (methyl), 2.1 (s, 3H) (acetyl), 4.1 (q, *J* = 6.83 Hz, 2H), 4.39–4.35 (m,1H) (methyne), 4.62 (dq, *J* = 4.25 Hz, 1H) (methylene), 6.86 (dq, *J* = 8.00 Hz, 1H) (aromatic), 7.11 (dt, *J* = 2.5 Hz, 1H) (aromatic), 7.25–7.17 (m, 2H) (aromatic), 7.38 (dq, *J* = 7.83 Hz, 1H) (aromatic), 7.83–7.77 (m, 1H) (aromatic), 7.98–7.94 (m, 1H) (aromatic), 8.1 (s, 3H) (hydroxyl); EIMS: M^+^ is probably very unstable and it goes to a fragmentation spontaneously. m/z (%) = 396 (0.09), 374 (0.9), 348 (0.5), 331 (0.9), 317 (5.3), 282 (8.8), 281 (12.3), 265 (9.6), 241 (1.8), 228 (26.3), 213 (0.9), 200 (48.2), 189 (1.8), 171 (8.8), 162 (93), 144 (7), 134 (5.3), 120 (100), 110 (58.8), 92 (74.6), 81 (8.8), 64 (35), 51 (11.4), 45 (3.5). TLC: *R*_*f*_ = 0.12 (hexane/chloroform/acetone/methanol = 5 : 3 : 2 : 1); Anal.: C_22 _H_20 _O_8_, (412), (C, H) (calcd/found): % C 64.07/64.44, % H 4.89/4.52.

**4-[1-(4-Hydroxy-2-Oxo-2H-Chromen-3-yl)-2-(Ethoxycarbonyl)-3-Oxobutyl]Benzoic Acid (11) [10]**

White crystals, m.p. 150–155°C. Purified after recrystalization from methanol. Yield: 28%, UV-VIS: *λ*_max⁡_ = 208, 282, 308 nm; FTIR (nujol): 3442, band 3300–2400, 1732.3, 1693.7, 1612.7, 1462.4, 1109, 846.3, 756.2 cm^−1^; ^1^H NMR (DMSO, 250 MHz): *δ* = 1.0 (t, *J* = 6.83 Hz, 3H) (methyl), 2.1 (s, 3H) (acetyl), 3.9 (q, *J* = 6.83 Hz, 2H) (methylene), 4.40–4.36 (m, 1H) (methyne), 4.62 (dq, *J* = 4.25 Hz, 1H) (methyne), 7.24–7.17 (m, 1H) (aromatic), 7.38 (dq, *J* = 7.83 Hz, 1H) (aromatic), 7.50–7.45 (m, 2H) (aromatic), 7.62–7.57 (m, 2H) (aromatic), 7.83–7.77 (m, 1H) (aromatic), 7.98–7.94 (m, 1H) (aromatic), 12.83 (s, 2H) (carboxylic); EIMS: m/z (%) = 424 (1.3, M^+^), 392 (0.4), 378 (17.5), 360 (1.8), 335 (48.2), 317 (19.3), 307 (9.6), 294 (44.7), 293 (34.2), 265 (6.1), 257 (50.9), 250 (22.8), 249 (100), 239 (5.3), 229 (1.8), 215 (12.3), 205 (1.8), 187 (2.6), 173 (2.6), 165 (4.4), 146 (2.6), 130 (6.1), 120 (19.3), 102 (6.1), 92 (30.7), 75 (6.1), 64 (8.8), 51 (2.6); TLC: *R*_*f*_ = 0.62 (hexane : chlorophorm : glacial acetic acid = 10 : 10 : 4); Anal. : C_23_H_20_O_8_, (424) (C, H) (calcd/found): % C 65.09/65.07, % H 4.75/4.9.

#### 4.1.5. The Michael Addition between 3-(4-Hydroxybenzylidene)-2,4-Pentanedione (SS-23) and 4-Hydroxycoumari

3-(4-Hydroxybenzylidene)-2,4-pentanedione (1.02 g, 0.005 mol) and 4-hydroxycoumarin (0.81 g, 0.005 mol) are mixed in slight excess of 4-hydroxycoumarin in 15–25 mL methanol. Piperidine (0.003 mol) as a basic agent is also added to the reagents. The reaction mixture is boiled and stirred for 60 hours under reflux. The reaction is controlled by TLC (hexane : chloroform : acetic acid = 10 : 10 : 4, hexane : chloroform : acetic acid = 10 : 10 : 2, hexane : acetone = 2 : 1). When the quantities of reagents are depleted, the heating was stopped. The residue from the reaction mixture was filtered off and was washed with hot water, in order to remove the 4-hydroxicoumarin which was not reacted. After that, the residue is dried at room temperature and recrystallized in acetone.

**Physical and Spectral Data**

**3-[(4-Hydroxy-2-Oxo-2H-Chromen-3-yl)(4-Hydroxyphenyl)Methyl]Pentane-2,4-Dione (12) [11]**

White crystals, m.p. 209–210°C. Yield 5%; ^1^H NMR (DMSO, 250 MHz): *δ* = 1.5 (s, 3H) (acetyl), 2.0 (s, 3H) (acetyl), 4.55–4.5 (m,1H) (methyne), 4.90 (dqui, *J* = 4.25 Hz, 1H) (methyne), 6.93–6.87 (m,2H) (aromatic), 7.2–7.17(m, 1H) (aromatic), 7.38 (dq, *J* = 7.83 Hz, 1H) (aromatic), 7.55–7.50 (m, 2H) (aromatic), 7.83–7.77 (m, 1H) (aromatic), 8.03–7.99 (m, 1H) (aromatic) 9.2 (s,2H) (hydroxyl); EIMS m/z (%) = 578 (0.8, M^+^), 523 (0.8), 495 (0.8), 467 (0.8), 439 (0.8), 409 (0.8), 395 (0.8), 382 (0.8), 368 (5.9), 354 (1.7), 3141 (2.5), 327 (0.8), 311 (6.7), 299 (1.7), 283 (5), 265 (58), 249 (16.8), 237 (6.7), 222 (1.7), 211 (2.5), 199 (1.3), 185(3.3), 165 (2.5), 146 (4.2), 121 (100), 100 (67.2), 85 (92.4), 65 (40.3), 53 (29.4); UV-Vis; *λ*_max⁡_ = 206,282 in ethanol; TLC: *R*_*f*_ = 0.38 (hexane/chloroform/acetone/methanol = 5 : 3 : 2 : 1), Anal.: C_21_H_18_O_6_ (366), (C, H) = (calcd/found): % C 68.85/68.36, % H 4.95/4.92.

### 4.2. Molecular Docking

All molecular docking calculations are performed witsh Maestro Macromodel Glide programs from Schrodinger package [18]. All of the structures (experimentally tested and new) are minimized by Macromodel program, using OPLS2005 force field and 5000 iterations. The X-ray structure of the enzyme HIV-1 protease, together with the inhibitor BEA369, is obtained from Protein Data Bank with PDB code 1EBY.

### 4.3. Cell Lines and Viruses

MT-4—a human lymphoblastoid suspension cell line, kindly provided by Gianfranco Pancino— Institute Pasteur, (Unite de Regulation des Infections Retrovirales, Paris, France) represent a classical model for experimental productive infection with HIV-1 III B strain and used as a routine target for study of effect of putative HIV inhibitors in cell culture [19]. 

As a source of HIV-1, supernatants of H9/HTLV III B line—a gift from Dr. R. Gallo (NIH, USA)— were used. Supernatants were collected and centrifuged to remove the cells, and virus stocks were prepared with known p24 antigen content (460 pg/mL, Murex HIV Antigen mAB test), RT activity (565.3 pg RT/mL, HS-Lenti RT Activity Kit, Cavidi, Sweden), and infectivity (2 × 10^6^ infectious virions/mL, microtiter infection assay) [20]. MT-4 and H9/HTLV III B cells were grown in RPMI 1640 supplemented with 10% FCS (invitrogen).

### 4.4. Cytotoxicity Tests and Antiviral Assays in Cell Culture

The compounds under study were first dissolved in DMSO and further diluted in cell growth medium without fetal serum. All the solutions were prepared *ex tempore*. 

The following parameters were studied: cytotoxic concentration 50—CC50, where possible (the concentration preventing death of 50% of MT-2 cells), maximal nontoxic concentration—MNC, and inhibitory concentration 50-IC50 (concentration inhibiting by 50% the viral replication). CC50 and MNC were detected by MTT uptake assay [12]. IC50 was studied on MT-4 cells by microtiter infection assay exploring the protection of cells from the cytopathic effect of HIV measured by MTT test [12]. Experiments under conditions of acute infection were performed in 96-well microplates with 6–8 parallels/experiment. Cell controls (MT-4 cells with medium only) and viral controls (virus infected MT-4 cells) were run with each experiment. For antivirus assays, HIV was added to each well to obtain multiplicity of infection 0.1 except the cell controls. Virus attachment was allowed for an hour at 37°C/5% CO_2_. The plates were incubated for 72–96 hours at 37°C/5% CO2. After that, MTT test was performed as described [12] and absorbance of viable cells was measured colorimetrically at A540 nm. For all experiments, the mean value of each column was calculated (only if the values in A540 did not differ in ±10%). For antiviral assays, the mean values of experimental and control rows were compared and the percentage of cells protected (cell survival) under the appropriate concentration of the substance was plotted against the concentration of the substance to obtain IC50. The cell survival (% of cell protection) was calculated according to the following formula:

(1) $$\begin{array}{cc} & \text{\%}\;\text{cell}\;\text{protection}\\ & \;=\frac{\text{A}540X-\text{A}540\;\text{Control}\;\text{HIV}}{\;\text{A}540\;\text{Cell}\;\text{Control}-\text{A}540\;\text{Control}\;\text{HIV}}\;\times 100, \end{array}$$

where *X* is the mean value of A540 of HIV-infected cells treated with appropriate concentration of the compound under study; control HIV is the mean value of A540 of HIV-infected cells without any compound added; cell control is the mean value of A540 of uninfected and untreated with inhibitor cells.

As referent substance, ABC (Abacavir well-known nucleoside reverse transcriptase inhibitor—NRTI) [21] and pepstatin were used.

#### 4.4.1. Endogenous RT Activity and Direct Effect of the Compounds on RT

It was tested by HS-Lenti Kit-RT assay (Cavidi, Sweden). The kit contains recombinant RT (rRT) as a standard, which makes possible RT quantitation. For endogenous RT assay, supernatants of HIV-1 infected/uninfected MT-4 cells after incubation with/without compounds were tested according to guidelines of manufacturer. The level of RT activity in supernatant was calculated (in pg/mL) from the HIV-1 rRT standard set in each kit. Direct effect of the compounds on rRT activity was measured with the same kit and aimed to prove RT as a target of antiviral action. Appropriate step dilutions of the inhibitor were prepared in control buffer and added to the reaction mixture. The reaction was run for 3 hours at 33°C.The RT activity of standard dilutions was compared to that where compounds were added or controls (where only incubation mixture without compounds was added). 

#### 4.4.2. Detection of Antiprotease Activity by Tests Using Native Viral PR

A method described earlier by Broglia et al., 2006 [19], for detection of recombinant protease activity was modified to use native viral protease [22]. As a source of native HIV-1 protease, again suspension of concentrated viral stock (50x) from chronically infected H9/HTLV IIIB cell supernatants was used. The lysis of viral particles and releasing of the active enzyme (protease) was performed using disrupting (lysis) buffer containing 2.5% Triton X-100 in phosphate buffer. The concentration of the tissue culture fluid containing the virus was done by ultracentrifugation in Biofuge Stratos, Heraeus, for 1 hour, at 4°C and 35000 rev/min. The pellet was resuspended to obtain 50x concentrate in disrupting buffer. 

The following reaction mixture was prepared for each experiment : 1000 *μ*L phosphate buffer (20 mM, pH 6.0); 20 *μ*L HIV protease substrate III (1 *μ*g/mL, 760 *μ*M; Bachem, Switzerland) in DMSO, prepared *ex tempore*; 20 *μ*L enzyme (stock HIV) taken from a solution containing 25 *μ*L disrupting (lysis) buffer + 100 *μ*L HIV-stock, incubated 40 min at 37°C prior to experiment. 

The HIV-1 PR activity was measured using direct spectrophotometric reading of the enzyme reaction substrate utilization at 300 nm, at room temperature and 1 cm pathlength, using T80 + UV-Vis spectrophotometer (PG instruments). The initial reaction velocity (V_0_) was adjusted to be 0.0020–0.0030 ΔAbs/min by varying the enzyme activity (viral concentration). The tested compound and the reference inhibitor (pepstatin used here) was added to the reaction mixture prior to enzyme, in order to perform the screening for inhibitory effect. IC50 was defined as the concentration of the compound tested that decreases the velocity of the reaction by 50% of the initial velocity without tested (reference) compound. 

## Figures and Tables

**Table 1:** Structural information and binding activity of the experimental compounds.

Experimental ligands	Substituents	IC_50 _ (*μ*M)	*G*-score	E-model
(**1**) 4-hydroxy-3-(1-phenoxypropyl)-2*H*-chromen-2-one	R = –C _3_H_6 _–O–C_6_H_5_, R_1_ = H	2.7	−7.04	−62.5
(**2**) 3-(3,4-dimethoxybenzyl)-4-hydroxy-2*H*-chromen-2-one	R = 3,4-dimethoxybenzyl, R_1_ = H	84	−6.17	−60.1
(**3**) 3-(3,4-dimethoxybenzyl)-4-hydroxy-8-methyl-2*H*-chromen-2-one	R = 3,4-dimethoxybenzyl, R_1_ = –CH_3_	23	−6.17	−59.5
(**4**) 3-benzyl-4-hydroxy-8-phenyl-2*H*-chromen-2-one	R = –benzyl, R_1_ = –phenyl	8.1	−6.27	−57.0
(**5**) 4-hydroxy-3-(3-oxo-1-phenylbutyl)-2H-chromen-2-one (Warfarin)	R = C_6_H_5_CHCH_2_COCH_3_ R_1_ = H	18	−6.77	−58.8

**Table 2:** Scoring function values of the tested compounds, according to the experimental ligands **(1)–(5)**.

Code	Ligand (**1**)		Ligand (**2**)		Ligand (**3**)		Ligand (**4**)		Ligand (**5**)	
	*G*-score	E-model	*G*-score	E-model	*G*-score	E-model	*G*-score	E-model	*G*-score	E-model
(**10**)	−6.96	−71.8	−7.31	−77.2	−6.90	−74.8	−6.18	−65.5	−7.08	−75.2
(**9**)	−5.73	−61.0	−7.00	−72.0	−7.00	−73.4	−5.44	−56.3	−7.02	−73.1
(**7**)	−5.63	−51.9	−7.46	−65.6	−6.04	−62.6	−5.97	−58.4	−7.85	−79.0
(**8**)	−6.23	−67.5	−6.26	−68.2	−6.11	−64.6	−6.11	−65.2	−6.31	−69.0
(**11**)	−5.78	−64.4	−6.04	−65.6	−5.94	−65.6	−5.84	−59.7	−6.00	−63.5
(**12**)	−5.36	−47.9	−6.13	−58.7	−6.42	−61.1	−5.75	−53.6	−6.41	−61.0

**Table 3:** Anti-HIV-1 activity in cell culture (MT-4 cells) and inhibition of HIV-1 protease activity.

Compound tested in (MNC-*μ*M)	Infection assay MTT- % cell survival	% Inhibition of HIV-1 protease activity
(**8**) (0.001)	0	0
(**9**) (0.0001)	0	0
(**10**) (0.0001)	30	16
(**11**) (0.0001)	0	0
(**7**) (0.001)	78	25
(**12**) (0.001)	0	0
Abacavir**^®^** (35 × 10^−6^ M)	98	Not tested
Pepstatin**^®^** (4.5 × 10^−5^ M)	Not tested	100

**^®^**reference inhibitors.
